# Variation in developmental rates is not linked to environmental unpredictability in annual killifishes

**DOI:** 10.1002/ece3.7632

**Published:** 2021-05-12

**Authors:** Piotr K. Rowiński, Will Sowersby, Joacim Näslund, Simon Eckerström‐Liedholm, Karl Gotthard, Björn Rogell

**Affiliations:** ^1^ Department of Zoology Stockholm University Stockholm Sweden; ^2^ Department of Biology Faculty of Science Osaka City University Osaka Japan; ^3^ Department of Aquatic Resources Institute of Freshwater Research Swedish University of Agricultural Sciences Drottningholm Sweden

**Keywords:** bet hedging, diapause, ephemeral habitats, maternal effects, plasticity, temperature response

## Abstract

Comparative evidence suggests that adaptive plasticity may evolve as a response to predictable environmental variation. However, less attention has been placed on unpredictable environmental variation, which is considered to affect evolutionary trajectories by increasing phenotypic variation (or bet hedging). Here, we examine the occurrence of bet hedging in egg developmental rates in seven species of annual killifish that originate from a gradient of variation in precipitation rates, under three treatment incubation temperatures (21, 23, and 25°C). In the wild, these species survive regular and seasonal habitat desiccation, as dormant eggs buried in the soil. At the onset of the rainy season, embryos must be sufficiently developed in order to hatch and complete their life cycle. We found substantial differences among species in both the mean and variation of egg development rates, as well as species‐specific plastic responses to incubation temperature. Yet, there was no clear relationship between variation in egg development time and variation in precipitation rate (environmental predictability). The exact cause of these differences therefore remains enigmatic, possibly depending on differences in other natural environmental conditions in addition to precipitation predictability. Hence, if species‐specific variances are adaptive, the relationship between development and variation in precipitation is complex and does not diverge in accordance with simple linear relationships.

## Graphical abstract

Variation in egg development rates differed substantially among seven species of annual killifish, which originate from a gradient of variation in precipitation rates. However, there was no clear linear relationship between variation in egg development time and variation in precipitation rate.

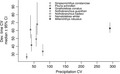

## 1 INTRODUCTION

Organisms can cope with fluctuations in environmental conditions by means of phenotypic plasticity or bet hedging (Crean & Marshall, 2009; Furness, Lee, et al., 2015; Simons, 2011). Yet, studies that simultaneously investigate both plasticity and bet hedging have largely been theoretical (e.g., Cooper & Kaplan, 1982; DeWitt & Langerhans, 2004; Marshall & Uller, 2007; Scheiner & Holt, 2012; Tufto, 2015, but see Bradford & Roff, 1993; Furness, Lee, et al., 2015; Marshall et al., 2008; Richter‐Boix et al., 2006; Simons, 2014; Shama, 2015). Understanding the evolution of these two adaptive strategies has become particularly critical in the face of global climate change, which is changing not only environments, but also the predictability of environmental conditions (Berg & Hall, 2015; Robeson, 2002; Thornton et al., 2014).

The evolution of plasticity and bet hedging requires variation in the fitness of individuals under different environmental conditions (i.e., phenotypes having differing environment optima; Simons, 2011). Phenotypic plasticity is an environment‐dependent trait expression; therefore, given genetic variation in reaction norms (i.e., genotypes differ in their associated phenotypes depending on environmental conditions; Simons, 2011), adaptive phenotypic plasticity is considered to evolve as a response to predictable environmental changes for which there are reliable cues (Ghalambor et al., 2007). However, phenotypic variation per se can be adaptive, as over a range of environmental conditions, by chance alone, the phenotypes of some individuals may be close to the environment‐dependent fitness optima (i.e., bet hedging; Crean & Marshall, 2009). When environmental conditions are either not predictable, or nontransducible into developmental regulators, the production of highly variable phenotypes will spread the risks associated with unsuitability to particular environmental conditions. Under this scenario, bet hedging is considered to be an adaptive strategy (Clauss & Venable, 2000; Crean & Marshall, 2009), although it is unclear how commonly these phenomena occurs.

The somewhat random apparent nature of bet hedging helps to ensure the survival of at least some offspring, by reducing among‐generation variation in reproductive success (Crean & Marshall, 2009). Bet hedging is considered to be a costly strategy with constant selection against a nontrivial part of the population that is not suited to current environmental conditions (Beaumont et al., 2009; Kussell & Leibler, 2005). Furthermore, while bet hedging can theoretically evolve in any trait, it is hypothesized to be particularly relevant in traits related to juvenile establishment, in highly fecund organisms that do not engage in parental care, and also in organisms that inhabit areas prone to rapid environmental fluctuations (e.g., “*r*‐selected” species, following MacArthur & Wilson, 1967).

In plants, comparative studies have repeatedly demonstrated bet hedging in seed dormancy (e.g., Evans et al., 2006; Philippi, 1993; Venable, 2007). For example, if there is a drought in any given year, variation in the duration of dormancy helps ensure that at least a subset of seeds will still likely germinate in the following years. In animals, bet hedging is observed when the optimal matching between phenology and environmental conditions is difficult to predict, such as the timing of diapause under winter or drought. Variation in developmental diapause duration during harsh periods (e.g., winter or dry seasons) has been reported in several animal taxa, including insects and fish (Furness, Lee, et al., 2015; Hopper, 2018). Importantly, the trait modified under a bet‐hedging scenario may not only concern developmental switching between different phenotypes (e.g., proportion of individuals that enter a diapause phase or not; Cáceres & Tessier, 2004; García‐Roger et al., 2014; Hopper, 2018; Seger & Brockmann, 1987), but also continuous traits, such as the length of diapause. In the short‐lived *Nothobranchius furzeri* killifish, for instance, egg development shows variation at multiple levels: whether they enter different diapause phases or not, duration of diapause, and timing of hatching (Furness, Lee, et al., 2015).

Bet hedging is an adaptive increase in phenotypic variation, and under a bet‐hedging scenario, trait variation is expected to correlate with environmental variation (Crean & Marshall, 2009). Species often inhabit environmental conditions that differ in both variability and predictability, with evidence from several studies suggesting that differences in bet‐hedging responses also occur among animal populations and species (García‐Roger et al., 2014; Krug, 2009; Marshall et al., 2008; Nevoux et al., 2010; Polačik et al. 2018). By comparing different species that inhabit environments, which vary in the predictability of certain environmental conditions or events, we can test for the potential presence of bet‐hedging strategies (Hopper, 2018; van Kleunen et al., 2014). Yet, questions remain regarding the adaptive nature of these patterns, as few studies have assessed differences across species or populations that occur over a gradient of environmental predictability (but see García‐Roger et al., 2014; Polačik, et al., 2016).

Here, we investigate one aspect of bet hedging by studying a continuous trait—mean and variation in the developmental time of eggs in seven species of annual killifishes (Cyprinodontiformes, Aplocheiloidei). These species inhabit ephemeral freshwater bodies in Africa and South/Central America, where both within‐ and between‐season conditions are often highly unpredictable (Furness, 2016; Genade et al., 2005; Inglima et al., 1981). Annual killifishes have evolved eggs capable of entering diapause several times independently as specific adaptations to inhabit ephemeral habitats. For instance, in these annual clades, eggs stay dormant (i.e., in diapause) buried in the substrate of dried‐out pools until the next wet season (Furness, Reznick, et al., 2015). However, even within species, the duration of egg diapause is variable and not always obligatory, meaning that one spawn of eggs may consist of both directly developing and diapausing eggs (of varying duration; Wourms, 1972b). This contrast in development time has been suggested to constitute a bet‐hedging strategy that maximizes fitness by spreading the risks associated with variability in the hydrological dynamics of ephemeral pools (Furness, Lee, et al., 2015; Polačik et al., 2014; Wourms, 1972b). Interestingly, variation in the duration of killifish diapause has been found to be both under maternal control and governed by plasticity during embryonic development (Furness, Lee, et al., 2015; Podrabsky et al., 2010; Polačik et al., 2016; Pri‐Tal et al., 2011).

Using annual killifishes, we measured the duration of egg development in a standardized laboratory setting. Furthermore, we correlated species‐specific means and variances of egg development time, with measurements of variation in precipitation rate, from the native ranges of each species during the onset of the rainy season. In the laboratory, eggs were reared under three different temperatures to assess the extent to which development time variation was stable across thermal conditions. The seven killifish species included in this study are representative of five of the annual clades that have independently transitioned from a nonseasonal to a seasonal life history (as indicated by the presence of type II diapause). These species were also chosen because they originate from a gradient of environmental conditions, with clear differences in the predictability of precipitation rates. We predicted that species that have evolved under conditions, which are relatively more unpredictable, will have larger variation in egg development rates, compared with species that have evolved in areas with more predictable environments. Alternatively, some species instead of relying on bet hedging strategy could try to match or prolong their development time until the environment is more probable to contain permanent water. In such case, we predicted that time of development would be correlated with variation in precipitation.

## 2 MATERIALS AND METHODS

### 2.1 Animal models

We used seven annual killifish species, *Gnatholebias zonatu*s (Myers 1935), *Millerichthys robustu*s (Miller and Hubbs 1974), *Nematolebias whitei* (Myers 1942), *Nothobranchius guentheri* (Pfeffer 1893), *No. kadleci* (Reichard 2010), *Pituna schindleri* (Costa 2007), and *Simpsonichthys constanciae* (Myers 1942), collected from 12 locations/populations (see Table 1), which are representative of five of the major independent evolutionary transitions between a nonannual and annual life cycle (Furness, Reznick, et al., 2015; *M. robustus* is situated in the Rivulus sensu stricto clade according to our preliminary phylogenetic analyses). The experimental procedures were approved by the Ethical Committee in Stockholm, Sweden (license N132/15).

**TABLE 1:** Population‐specific data with population‐specific and species‐wide coefficients of variation (CVs) across their distributions, in among‐year precipitation during the rainy season months

Species	Population, collection year	Climate data coordinates	Rainy season months	Population‐specific precipitation mean (mm)	Species‐wide precipitation mean (mm)	Population‐specific precipitation CV (%)	Species‐wide precipitation CV (%)
Gnatholebias zonatus	Finca Las Palmas, Colombia, 2014	4.004N:73.167W	Mar, Apr, May	311	242	40.9	42
Gnatholebias zonatus	Las Mercedes, Venezuela, 2014	9.10N:66.39W	May, Jun, Jul	173		44.3	
Millerichthys robustus	Tlacotalpan, Veracruz, Mexico, 2017	18.627N:95.648W	Jun, Jul, Aug	434	434	257.9	258
Nematolebias whitei^a^	Buzios, Brazil: *first rainy season*	22.771S:41.944W	Mar, Apr, May	67	77	62.8	78
Nematolebias whitei^a^	Buzios, Brazil: *second rainy season*	22.771S:41.944W	Oct, Nov, Dec	87		92.7	
Nothobranchius guentheri	Zanzibar 2014	5.017S:39.750E	Mar, Apr, May	352	317	89.5	57
Nothobranchius guentheri	Zanzibar 2014	6.025S:39.328E	Mar, Apr, May	311		50	
Nothobranchius guentheri	Zanzibar 2014	6.167S:39.367E	Mar, Apr, May	288		33.2	
Nothobranchius kadleci	Pungwe, Moçambique, 2012	19.291S:34.231E	Dec, Jan, Feb	170	181	54	60
Nothobranchius kadleci	Nhamatanda, Moçambique, 2011	20.688S:34.107E	Dec, Jan, Feb	201		62.2	
Nothobranchius kadleci	Save, Gorongose, Moçambique, 2008	21.015S:34.463E	Dec, Jan, Feb	171		65.5	
Pituna schindleri	União, Piauí, Brazil	4.674S:42.005W	Dec, Jan, Feb	203	203	39.2	39
Simpsonichthys constanciae	Barra de Sao Joao, Brazil, 1995	22.030S:42.020W	Oct, Nov, Dec	186	186	27.3	27

^a^ In case of *N*.* whitei*, data concerning the same population are presented in separate rows for the two rainy seasons.

### 2.2 Rearing of parental generation

The parental generation was hatched from eggs sourced from dedicated hobbyists, or from our own laboratory‐housed breeding groups. The parental generation was hatched under standardized conditions and raised individually in 0.75‐L plastic containers with ramshorn snails (Planorbidae) to consume uneaten food, and java moss (*Taxiphyllum barbieri*) for shelter. Fish were all fed (three times/day during weekdays and one time/day during weekends) with freshly hatched *Artemia salina* nauplii and reared under standardized conditions (ambient temperature 28°C, average water temperature 24.2°C ± 0.65 *SD*). Tap water (KH = 4, GH = 7, pH = 7.5), with the addition of Jbl Biotopol (JBL GmbH & Co) water conditioner, was used to fill aquaria. The parental fish were pooled into groups of 3–4 individuals at 1 week of age, in one 0.75‐L plastic box. When they then reached 1 cm in total length, they were transferred to 13‐L tanks and fed with a mixture of defrosted Chironomid larvae and live *Artemia salina* nauplii. The 13‐L tanks were furnished with gravel, an empty terracotta flowerpot, and a yarn mop. Water quality was maintained with an air‐driven sponge filter, and twice‐weekly 80% water changes in the 0.75‐L plastic containers and weekly 25% water changes in the larger 13‐L tanks.

### 2.3 Breeding procedures

When females were noticeably mature, that is, with a rounded egg‐filled body cavity, they were paired with a randomly chosen male and placed together in a 13‐L spawning tank for a period of 2–3 months. Female annual killifish either bury or disperse eggs over a substrate; therefore, a 0.75‐L plastic container, filled with either glass beads (Sargenta AB) or coco peat (Exo Terra), was added into each breeding tank to provide substrate for egg laying. We found that glass beads facilitated more efficient egg retrieval, but these were not readily accepted by all species as an appropriate breeding substrate, so those species (*n* = 3) were supplied exclusively with boxes filled with coco peat. In most species (*n* = 6), some males were particularly aggressive and were therefore grouped together with 2–4 females to dilute aggression among a higher number of females. Females were regularly switched into different tanks with a different male, residing in each for 2–3 months. Eggs were gathered weekly, either by sieving the glass beads through a net, or by laying the coco peat on a white plastic board and inspecting thoroughly for the eggs.

### 2.4 Experimental treatment of the offspring generation

We included different temperature treatments during egg incubation to assess whether developmental times are congruent across different thermal regimes. This is in line with Furness, Lee, et al. (2015), who investigated bet hedging and developmental plasticity in *No. furzeri*, and found that percentage of eggs entering diapause differed depending on rearing temperature. However, while Furness, Lee, et al. (2015) used a rather broad range of rearing temperatures, we used a range of temperatures that is likely within the range of what embryos of all the species would encounter in the wild during dry season. In this regard, fertilized eggs from each male tank (per female or female group) were evenly partitioned into three different egg incubators (Lucky Reptile Herp Nursery II; Import Export Peter Hoch GmbH) set to 21°C (± 0.15 SD), 23°C (± 0.44 SD), and 25°C (± 0.33 SD). All adult/parental fish were kept under a 12:12‐hr light regime, and while the developing embryos were also kept in the same laboratory, they were housed in incubators and therefore not exposed to direct light. There are several recognized methods for incubating killifish eggs (see Polačik et al., 2016), but to maximize the standardization of rearing conditions and to avoid artificially increased within‐species variation, we chose to incubate eggs submerged in a Yamamoto solution (17 mM NaCl, 2.7 mM KCl, 2.5 mM CaCl_2_, pH set to 7 with NaHCO_3_; Valenzano et al., 2009 after Rembold et al., 2006; also successfully used by Furness, 2016; Furness, Lee, et al., 2015, Furness, Reznick, et al., 2015). In addition, two drops of 6.25 mM methylene blue and 5.33 mM acriflavine solution were added per 1 L of Yamamoto solution, to prevent the occurrence of fungus and bacterial infections. For incubation, each embryo was transferred to a separate well containing the incubation medium, on a 24‐well tray (TC Plate Standard F; Sarstedt AG & Co. KG). The Yamamoto solution was changed twice a week to ensure clean and stable solution conditions.

### 2.5 Data collection

Killifishes can have up to three distinct embryonic diapause stages, referred to as type I (developmental arrest during early development, dispersed cell phase), type II (38 somites present, beginning of organ development), and type III (embryo developed and able to hatch; Furness, Reznick, et al., 2015; Wourms, 1972a). During type II diapause, embryos are particularly resistant to dehydration stress, and this stage is only observed in annual killifish (Furness, 2016; Furness, Reznick et al. 2015; Podrabsky et al., 2015). Type II diapause facilitates survival in harsh environmental conditions and can last for many months, the time until the end of type II diapause is hence considered an important time point for survival during the dry season (Furness, Lee, et al., 2015; Podrabsky et al., 2015). Moreover, it may be beneficial for the embryos to stay in type II diapause until ponds completely fill, in the case that ponds redry after an initial period of rain. We visually inspected embryos weekly with a magnifying glass for the appearance of pigmented eyes, which become pigmented after the type II diapause phase (Wourms, 1972a). The period between the egg‐laying date and the appearance of pigmented eyes was then used as a proxy for developmental time. In total, we collected 2,567 eggs of which 24% (*N* = 614) survived until the eye pigmentation stage. The majority of mortality occurred shortly after egg collection, with half of the mortality occurring during the first week of incubation, presumably due to being unfertilized or inflicted with minor damage during collection, which may have exposed the embryos to oomycete (Saprolegniaceae) infections. At the end of experimental period (which ran between July 2017 and June 2019), 21 embryos did not show eye development. These undeveloped embryos, which were evenly distributed across the temperature treatments, belonged to two species, *G*. *zonatus* and *P*. *schindleri*, and were excluded from the analysis. We consider these exclusions very unlikely to have influenced our results, as they represented less than 1% of the total embryos included in our study, and around 5% of embryos collected for each of these two species.

### 2.6 Estimation of precipitation variability

To assess the influence of precipitation variability on both embryo development time and variation in development time, we identified the exact coordinates of the collection localities of our laboratory species from the killi‐data.org archive (Huber et al., 2016), and retrieved site‐specific climate data for each locality from the Local Climate Estimator software (New_LocClim, average length of time series is 50 years; FAO, 2018). The following variables were collected: (a) mean precipitation for the three first months of the rainy season among years and (b) standard deviations in precipitation for the three first months of the rainy season among years. We chose these specific parameters, as precipitation predictability during the rainy season should be key to complete pond‐filling and, hence, crucial for embryo survival. We reasoned that high variation in precipitation rates during the early rainy season may result in ponds only partially filling. Partial filling may be enough to induce killifish eggs to hatch, but possibly not provide enough time to complete their entire life cycle, as a partially filled pool might desiccate quickly in times of drought resulting in all fish from a given generation dying. We used a 3‐month period because embryos often hatch after at least 2 months after first rain (Domínguez‐Castanedo et al., 2017; Polačik et al., 2011; Watters, 2009). For each species, the first month of a rainy season was assumed to be the month when precipitation increased, following the dry season. The dry season was considered as a within‐year period of low precipitation, with average precipitation falling under 60 mm/month (Peel et al., 2007) for at least 1 month. In the case of *Ne. whitei*, the differentiation between dry and rainy season was not as obvious as other species and indicated the existence of two separated rainy seasons. Hence, we averaged precipitation data for these two periods (Table 1). When our laboratory populations originated from more than one collection locality, we averaged the climate data for these populations (Table 1). The averaged climate data are therefore representative for the species, as different populations of the same species used in this study typically inhabit similar environments (Table 1). To ensure a large enough sample size across species, our study was conducted at the species level and did not consider population‐level differences any further.

We found that there are large divergences among the species in mean rainfall. As variance scales to the mean, we calculated species‐specific coefficients of variation (CV) for monthly precipitation (average length of data series being 50 years), as the standard deviations multiplied by 100, divided by the mean, and averaged the monthly CVs for the first 3 months of the rainy season.

### 2.7 Statistical models

To examine the sources of variation in egg development time, we first fit two separate intercept‐only models. The first model was fit on a subset of the whole data, when female identity was known (i.e., data from breeding tanks containing only one female, model 1a; Table 2), and included species, male, and female, as random effects. The second model was fit to the full dataset, including those observations where female identity was unknown (breeding tanks with both single females and female groups, model 1b; Table 2), and included species, male, and female/female group, as random effects. Of these models, the former model gave us an opportunity to estimate female effects, while the later model provided better estimates of species effects, given the larger dataset. We then calculated medians and 95% confidence intervals of male, female/female group, within‐, and among‐species posterior distributions of variation.

**TABLE 2:** List of the models

Model no.	Model testing	Model formula
1a	Female effects, run on the data including single‐female tanks only	development time ~1, random = ~spec + male + female
1b	Species and male effects, run on full data	development time ~1, random = ~spec + male + female
2	Differences among species means and variances, pooled temperature treatments	development time ~1, random = ~spec, rcov = ~idh(spec):units
3	Differences among species means and variances, 21°C	development time ~1, random = ~spec, rcov = ~idh(spec):units
4	Differences among species means and variances, 23°C	development time ~ 1, random = ~spec, rcov = ~idh(spec):units
5	Differences among species means and variances, 25°C	development time ~ 1, random = ~spec, rcov = ~idh(spec):units

In order to (a) correlate environmental predictability with means and variances of development time, and (b) compare among‐temperature means and variances of development time, we first ran four intercept‐only models, one model per temperature treatment, and one with pooled temperatures. All models included species as a random effect and allowed for separate residual variance among species (models 2–5, Table 2). From these models, we obtained full posterior distributions for species‐, and temperature‐specific means and variances of development time, which allowed for the calculation of posterior distributions for CVs in developmental times. The posterior distributions for the CVs were calculated as the square root of posterior distribution for variances, multiplied by 100, and divided by the posterior distribution for the mean. In order to assess the significance of species‐specific responses to temperature, we calculated the differences among the three temperature‐specific posterior distributions for each species (Figures 2 and 4). Significance was determined as a lack of overlap with 0.

Finally, to test whether variation in development time increased with increasing variation in precipitation, as would be expected under a bet‐hedging scenario, we modeled CVs of development time as a response variable and a vector of precipitations CVs as an explanatory variable. This was done for the full posterior distribution of CV (*N*_iterations_ = 1,000) to account for uncertainties in the estimates. In addition, for each iteration, we bootstrapped over species, in order to account for uncertainty associated with the included species (where, e.g., *M. robustus* could be classified as an outlier). This approach yielded posterior distributions of the slope of the regression, where significance was determined as a lack of overlap with 0. We used the same approach to examine whether means of development time were associated with precipitations CVs.

### 2.8 Potentially confounding factors

Maternal age has previously been found to affect egg development time in killifishes (Podrabsky et al., 2010; Polačik, Smith, et al., 2016; Pri‐Tal et al., 2011). Therefore, we wanted to exclude the sources of maternal effects that could be viewed as experimental artifacts. Specifically, as pairing was performed over an extended period, the age of parental fish differed across different full‐sib families, which previously has been found to influence embryo development time (Podrabsky et al., 2010). Moreover, while our intention was to create full‐sib offspring families, some males were more aggressive and multiple females were required in the mating tanks, which could potentially influence egg developmental trajectories. Hence, we initially ran a model with female age (continuous variable) and female group rearing (factor with two levels, single and group) as fixed effects. Species, male, and the female/female group identity were added as random effects, in order to assess whether female age or group/single female breeding could constitute a source of maternal effects, and influence the results. However, as these factors were nonsignificant, they were not included in any of our inferential models.

### 2.9 Model evaluation

Data were analyzed using the Bayesian mixed‐effects models (MCMCglmm package for R; Hadfield, 2010), in R version 3.4.4 (R Development Core Team, 2015). In all models, we used flat priors for the fixed effects and locally noninformative priors for the random effects, a burn‐in of 5 × 10^4^ followed by at least 1 × 10^6^ iterations, and a thinning interval of 1,000, which resulted in effective sampling size of >1,000 iterations. We diagnosed posterior distributions and model convergence by running three parallel chains using the Gelman–Rubin convergence criterion (Brooks & Gelman, 1998); the upper 97.5 quantile of the Gelman–Rubin test statistic was below 1.2 in all cases. All autocorrelations were within the interval −0.1 and 0.1.

## 3 RESULTS

We found that most of the variation in development time was structured within species, as the residual variation accounted for 70% (95% CI: 36, 85) of the total variation (averaged across species). Among species variation accounted for 28% (95% CI: 12, 63) of the total variation, while male and female influences were negligible (male <1%, female <1%, and female/female group <1%).

### 3.1 Species‐ and temperature‐specific length of development time

**FIGURE 1:**
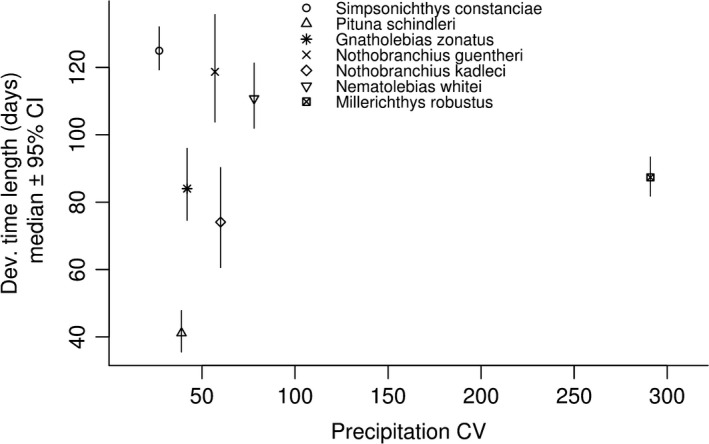
Species‐specific medians of posterior distributions of development time length, and their 95% credibility intervals (y‐axis), against precipitation CV (x‐axis)

**FIGURE 2:**
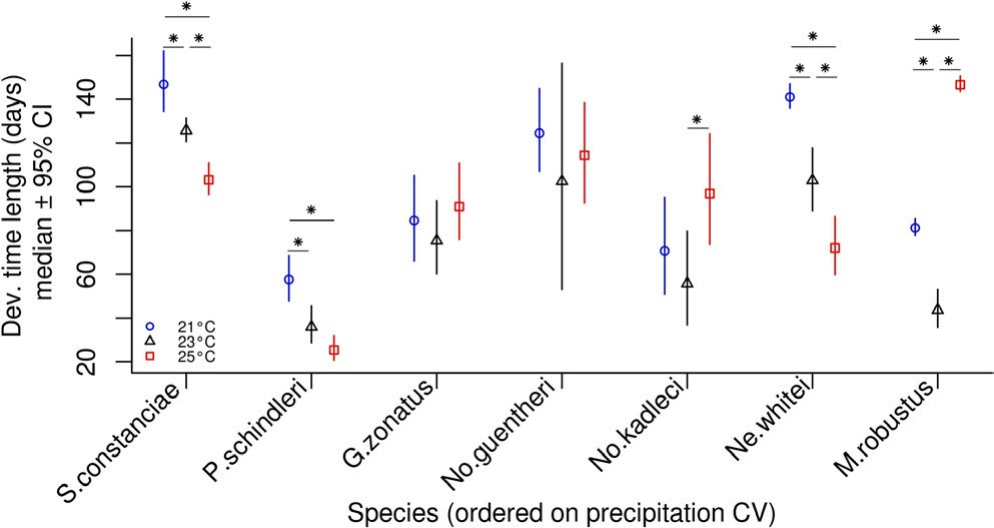
Species‐ and rearing temperature‐specific medians of posterior distributions of development time length, and their 95% credibility intervals (y‐axis). Species are ordered on a categorical x‐axis scale, according to precipitation CV values, from the lowest (left) to the highest (right), for clarity of the results. Star symbols indicate significant within‐species differences between the temperature treatment groups

Development time differed significantly among species (Figure 1), and among different rearing temperatures within species (Figure 2), as indicated by nonoverlapping 95% CIs of the posterior distributions. However, there was no clear linear relationship between development time and precipitation CV (*β* = −0.0048; 95% CI: −0.82, 1.65; Figure 1). Furthermore, there was no general relationship between development time and rearing temperature, although in a subset of species, *S. constanciae, P. schindleri,* and *Ne. whitei*, higher temperature corresponded with shorter development times (Figure 2).

### 3.2 Species‐ and temperature‐specific variation in development time

**FIGURE 3:**
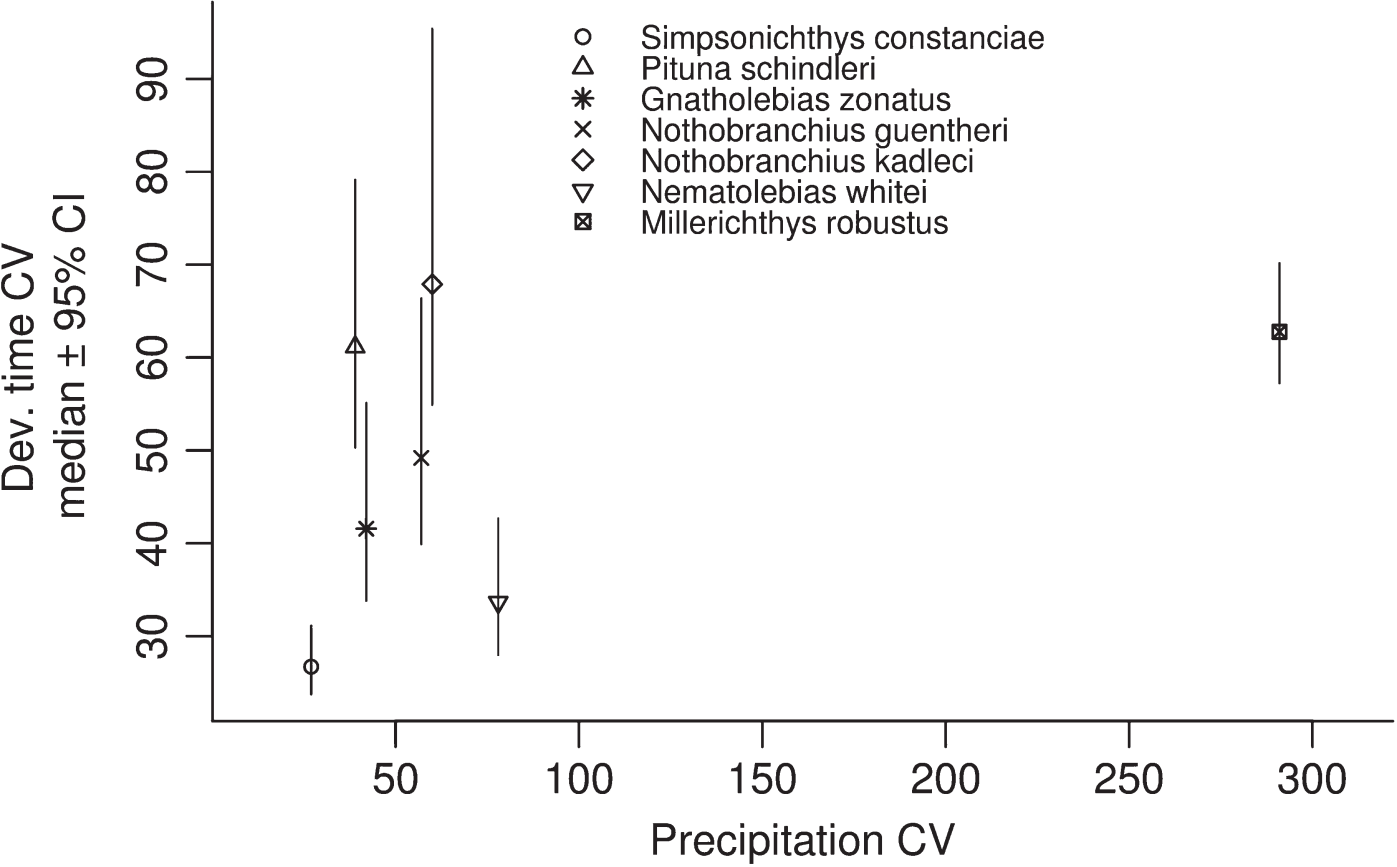
Medians of posterior distributions of species‐specific coefficients of variation in development time, and their 95% credibility intervals (y‐axis), against precipitation CV values (x‐axis)

**FIGURE 4:**
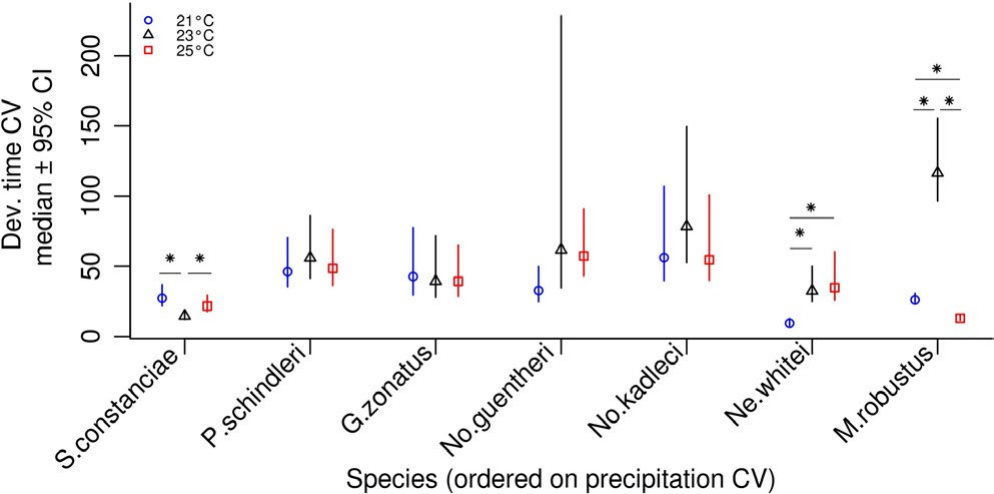
Medians of posterior distributions of species‐ and temperature‐specific coefficients of variation in development time, and their 95% credibility intervals (y‐axis). Species are ordered on a categorical x‐axis scale, according to precipitation CV values, from the lowest (left) to the highest (right), for clarity of the results. Star symbols indicate significant within‐species differences between the temperature treatment groups

Development time CVs differed significantly among species (Figure 3), and among different rearing temperatures within species (Figure 4; *S. constanciae*, *Ne. whitei*, *M. robustus*), as indicated by nonoverlapping 95% CIs of the posterior distributions. There was no clear linear relationship between development time CV and precipitation CV (*β* = 0.077; 95% CI: −0.56, 1.13; Figure 3). There was no detectable general relationship between development time CV and rearing temperature (Figure 4).

## 4 DISCUSSION

We found substantial among‐species differences in both the mean and the variation of egg development time in annual killifish species, which originate from environments along a gradient of precipitation variability. Under a precipitation‐driven bet‐hedging scenario, we expected species from more unpredictable environments to have higher variation in embryo development times (Crean & Marshall, 2009). However, we did not find any general relationship between variation in precipitation and variation in development time across species. Moreover, in several species, both the mean and the variation of development time were dependent on rearing temperature. The lack of association between variation in development time and environmental variation suggests that bet hedging either may not be an important mechanism for persisting in these ephemeral habitats, or that other environmental factors also influence developmental times. Hence, if species‐specific variances are adaptive, the relationship between development and variation in precipitation is complex, and does not diverge in accordance with simple linear relationships. In addition, it is possible that studying a broader set of traits, including entrance into different types of diapauses and timing of hatching, may be needed to better understand the evolution of bet hedging in killifishes.

In contrast to our results, comparative analyses have shown that annual desert plant species occurring in areas with unpredictable rates of precipitation produce seeds with highly variable germination times (Venable, 2007; see also Evans & Dennehy, 2005). We did not track if embryos entered different types of diapauses, but rather used the time until eye development as a proxy for general inclination toward longer development times through diapause. Therefore, development time in the killifish system could be viewed as analogous to the example of seed banks in plants. While the seasonal pond filling period, and thus precipitation, is crucial for the survival of annual killifishes (Domínguez‐Castanedo et al., 2017; Polačik, et al., 2016; Watters, 2009), we did not find any clear relationship between precipitation predictability and variation in embryo development time. Our results are hence similar to a recent study that did not find any differences in development times among eight populations of two closely related annual *Nothobranchius* killifish species (*No. furzeri* and *No. kadleci*; Polačik, et al., 2016). Together, these results suggest that other factors likely also contribute to driving differences in egg development patterns across species. Even though we found no evidence for bet hedging, our results do still implicate a link between development and environmental conditions. For example, *Ne*. *whitei* and *S. constanciae* co‐occur in areas with two rainy seasons per year (Costa, 2012), and despite key differences in other traits between these two species (e.g., egg size and growth rate; Eckerström‐Liedholm et al., 2017; Huber et al., 2016; Sowersby et al., 2019), egg development time was similar, both in terms of the variation (relatively low), mean (relatively long), and sensitivity to incubation temperature. These phenotypic similarities among two co‐occurring species suggest that developmental time might be shaped more by the natural conditions under which these species occur.

In a bet‐hedging scenario, there will be constant selection against a nontrivial part of the population that is not suited to current environmental conditions (Beaumont et al. 2009; Kussell & Leibler, 2005). However, a lack of bet‐hedging, particularly in unpredictable environmental conditions, may also be costly. For example, without a bet‐hedging strategy, all offspring may be ill‐equipped to cope under current environmental conditions. Variation in one key trait, development time, may therefore evolve as an evolutionary trade‐off between the costs and benefits of employing a bet‐hedging strategy. Hence, one potential explanation for our results may be that different species exhibit different solutions to this trade‐off, by either trying to match the rainy season (high risk–high payoff strategy), or by having large variation (low risk–low payoff strategy) in development rates. Either scenario could yield the pattern we observed, with large among‐species differences in egg development rates, which were not necessarily linked to environmental predictability.

Plastic responses and adjustments in trait variance are important for facilitating adaptation, particularly as a warming climate changes both the environment and its predictability (Berg & Hall, 2015; Robeson, 2002; Thornton et al., 2014). Species that utilize bet‐hedging strategies are hypothesized to be better adapted to cope with increased environmental variation including climate change, as they use a risk‐minimizing strategy (Childs et al., 2010). In our study, species with higher variation in development time (e.g., *P. schindleri*, *No. kadleci*, and *M. robustus*) may be predisposed to cope better with future environmental changes; however, at the same time they are likely to suffer significant costs, as a large proportion of a female's eggs are unlikely to develop in any particular season.

In addition to differences among species in variation in embryo development time, we found that both the mean and the variation in development time were affected by rearing temperature. In regard to mean development time, this result was as expected, as thermal plasticity is pronounced in ectotherms, and generally increases the speed of biological processes (Roff, 2002; for killifish diapause specifically, see Furness, Lee, et al., 2015; Levels & Denucé, 1988; Podrabsky et al., 2010). For three of the species in our study (*S. constanciae*, *P. schindleri*, and *Ne. whitei*), we found this expected pattern of typically ectotherm development, with higher rearing temperatures speeding‐up developmental times.

For some species, we found patterns between incubation temperature and mean embryo development time, although the patterns of temperature‐mediated changes in the variation of developmental time (*coefficient of variation*) were less clear. However, there were some significant differences among the variations estimated for each thermal incubation regime. Differences among the coefficients of within‐species variation, across thermal regimes, tended to occur in species from more temperate areas with higher temperature variation (South: *S*.* constanciae*, *Ne. whitei*, North: *M*.* robustus*). Yet, across these species there was no clear directionality in how the different temperatures affected the coefficient of variation for development time. For example, while *M. robustus* had the highest variation in the intermediate incubation temperature (23°C), *S. constanciae* had the lowest variation in this intermediate temperature. Our results hence suggest that evolution under more temperate thermal conditions both increases the evolution of thermal plasticity and decreases the canalization of traits. However, it is difficult to make any inferences on the adaptive value of increased variations across the different temperatures.

Factors such as maternal age (Podrabsky et al., 2010), egg‐laying order (Polačik, Smith, et al., 2016), and hormonal levels in mothers (Pri‐Tal et al., 2011) have been reported to also influence variance in developmental rates of annual killifish embryos. We found no evidence that any maternal factors influenced developmental times in our study (maternal age and group/single breeding). We did not have sufficient statistical power (due to low number of eggs per family) to test whether females or males differed in terms of variation in the development time of the eggs/embryos they produced. Species variance did account for a large part of the variation in the model, with little influence of male and female effects, suggesting that species have instead evolved different development times due to differences in their natural environment. In addition to maternal influence, several other environmental factors have been found to induce plastic effects on the length of embryonic development, such as temperature (Furness, Lee, et al., 2015; Levels & Denucé, 1988; Podrabsky et al., 2010), photoperiod (Furness, Lee, et al., 2015; Levels & Denucé, 1988; Podrabsky & Hand, 1999), hypoxia (Inglima et al., 1981), and the presence of other fish in aquaria with developing embryos (Inglima et al., 1981; Levels et al., 1986). While these factors are mostly standardized in our experiment, we cannot exclude the possibility that they affected our results. In our study, we kept developing embryos in water (solution), which allowed for highly standardized conditions but could decrease development times (Polačik, Blažek, et al., 2016). Specifically, we cannot exclude the possibility that species differ in how they respond to developing in water (opposed to developing buried in substrate), which could in part explain our results. However, raising the embryos in a substrate could likely artificially increase variation in development times due to differences in humidity, as controlling for humidity for each separate embryo simultaneously was not feasible due to logistical reasons. We also used captive‐bred fish populations, which may mean that some of the species could be relatively inbred. However, all but one species has been collected from the wild within the past 11 years, suggesting that inbreeding effects have had a relatively short time to accumulate (Table 1). As inbreeding is considered to decrease genetic variation, we note that virtually all species showed considerable levels of variation in development times (CV > 20). Hence, we do not believe that inbreeding has driven the patterns observed in our results. Finally, it is possible that other noncontinuous traits need to be studied simultaneously with development time in order to get a better picture of bet hedging in killifish. For example, although a previous study on two *Nothobranchius* species did not find among‐population differences in development times, it found a significantly higher proportion of short‐developing embryos in fish from more arid regions (Polačik, et al., 2016). Therefore, comparative studies investigating a full spectrum of bet‐hedging traits, such as whether they enter different types of diapause or not, how long development is, and what the timing of hatching is, may be a fruitful avenue for future research.

In conclusion, we found that means and variation in developmental times differed among seven annual killifish species, irrespective of environmental predictability. Moreover, we found among‐ and within‐species differences in response to temperature treatments, with more pronounced changes in developmental time than developmental time CV, and with three species from more temperate areas being more plastic, compared with species from more tropical areas. Although we were unable to pinpoint the exact causes of these observed differences, we suspect that a combination of environmental factors plays an important but as‐of‐yet unidentified role, in influencing embryo development times. Therefore, it will be important to investigate factors potentially influencing embryo development times encountered in their natural environment, especially with climate change further increasing environmental unpredictability.

## CONFLICT OF INTEREST

The authors declare no conflicts of interests.

## AUTHOR CONTRIBUTION

**Piotr Kosma Rowinski:** Conceptualization (equal); Data curation (lead); Formal analysis (equal); Investigation (lead); Methodology (equal); Software (lead); Visualization (lead); Writing‐original draft (equal); Writing‐review & editing (equal). **Will Sowersby:** Investigation (supporting); Methodology (supporting); Supervision (supporting); Validation (equal); Writing‐review & editing (equal). **Joacim Näslund:** Methodology (supporting); Supervision (supporting); Validation (equal); Writing‐review & editing (equal). **Simon Eckerström‐Liedholm:** Methodology (supporting); Validation (supporting); Writing‐review & editing (supporting). **Karl Gotthard:** Methodology (supporting); Project administration (supporting); Supervision (supporting); Validation (equal); Writing‐review & editing (equal). **Björn Rogell:** Conceptualization (equal); Formal analysis (equal); Funding acquisition (lead); Methodology (equal); Project administration (lead); Resources (lead); Supervision (lead); Validation (lead); Writing‐original draft (equal); Writing‐review & editing (equal).

## ETHICAL APPROVAL

The experimental procedures were approved by the Ethical Committee in Stockholm, Sweden (license N132/15).

## Data Availability

The experimental data have been deposited to the Dryad archive (https://doi.org/10.5061/dryad.8sf7m0cn0).
